# ICG-001 affects DRP1 activity and ER stress correlative with its anti-proliferative effect

**DOI:** 10.18632/oncotarget.22264

**Published:** 2017-11-01

**Authors:** Heidi Zinecker, Djamila Ouaret, Daniel Ebner, Moritz M. Gaidt, Steve Taylor, Anna Aulicino, Marta Jagielowicz, Veit Hornung, Alison Simmons

**Affiliations:** ^1^ MRC Human Immunology Unit, Weatherall Institute of Molecular Medicine, University of Oxford, John Radcliffe Hospital, Oxford, OX3 9DS, United Kingdom; ^2^ Department of Oncology, Cancer and Immunogenetics Laboratory, Weatherall Institute of Molecular Medicine, University of Oxford, John Radcliffe Hospital, Oxford, OX3 9DS, United Kingdom; ^3^ Target Discovery Institute, University of Oxford, Oxford, OX3 7FZ, United Kingdom; ^4^ Gene Center and Department of Biochemistry, Ludwig-Maximilians-Universität München, Munich, 81377, Germany; ^5^ Computational Biology Research Group, Weatherall Institute of Molecular Medicine, University of Oxford, John Radcliffe Hospital, Oxford, OX3 9DS, United Kingdom

**Keywords:** DRP1, drug screening, ICG-001, ER stress, colorectal cancer

## Abstract

Mitochondria form a highly dynamic network driven by opposing scission and fusion events. DRP1 is an essential modulator of mitochondrial fission and dynamics within mammalian cells. Its fission activity is regulated by posttranslational modifications such as activating phosphorylation at serine 616. DRP1 activity has recently been implicated as being dysregulated in numerous human disorders such as cancer and neurodegenerative diseases. Here we describe the development of a cell-based screening assay to detect DRP1 activation. We utilized this to undertake focused compound library screening and identified potent modulators that affected DRP1 activity including ICG-001, which is described as WNT/β-catenin signaling inhibitor. Our findings elucidate novel details about ICG-001’s mechanism of action (MOA) in mediating anti-proliferative activity. We show ICG-001 both inhibits mitochondrial fission and activates an early endoplasmic reticulum (ER) stress response to induce cell death in susceptible colorectal cancer cell lines.

## INTRODUCTION

Mitochondria form a strongly connected, highly dynamic network driven by opposing scission and fusion processes that control their function and distribution. The mitochondrial fission protein DRP1 belongs to a protein superfamily of large guanosine triphosphate phosphohydrolases (GTPases) that includes classical dynamins as well as the mitochondrial fusion proteins optic atrophy 1 (OPA1), mitofusin 1 (MFN1) and mitofusin 2 (MFN2) [1]. DRP1 is mainly a cytosolic protein, which is recruited to the outer mitochondria membrane via its receptor proteins FIS1, MFF, MiD49 and/or MiD51 [2, 3]. At mitochondria-ER contact sites DRP1 oligomerizes into ring-like structures, and subsequent mitochondrial constriction is driven by DRP1-catalyzed GTP hydrolysis. Finally, in coordinated manner with another GTPase, dynamin 2, the scission process of mitochondria takes place [4, 5].

Posttranslational modifications such as ubiquitination, SUMOylation, O-GlcNAcylation and phosphorylation have been demonstrated to be key for the regulation and function of DRP1 [6–8]. DRP1 comprises two critical phosphorylation sites, phosphorylation of serine 616 is activating and DRP1^Ser637^ has been reported as inhibitory. Indeed, phosphorylation of serine 637 by cAMP-dependent protein kinase (PKA) inhibits DRP1 enzyme activity [9]. Similarly, calcineurin catalyzed dephosphorylation results in increased mitochondrial fragmentation [9, 10]. Phosphorylation of serine 616 catalyzed by Cdk1/cyclin B occurs during mitosis in the course of G2/M phase transition ensuring proper distribution of mitochondria during cell division by increased fragmentation [11]. Further, DRP1^Ser616^ phosphorylation depending on ERK activation promotes cellular transformation and tumor progression, and controls reprogramming of somatic cells to pluripotency [12–14]. Mitochondrial dysfunction affects numerous pivotal, cellular mechanisms such as metabolism, apoptosis, autophagy and cell cycle regulation [15]. Intriguingly, a plethora of studies have demonstrated a role of mitochondrial dynamics and morphology for the treatment of cancer, obesity, diabetes, cardiac diseases as well as neuronal injuries and neurological disorders including diseases such as Alzheimer’s, Huntington’s and Parkinson’s disease [16–18]. Recently, it has been reported that impaired mitochondrial fusion and *vice versa* increased mitochondrial fission activity is associated with increased proliferation leading to enhanced cancer growth and tumorigenesis [12, 13, 19]. Amplified DRP1 expression and/or phosphorylation was demonstrated in multiple cancer cell types such as breast cancer, lung cancer, and glioblastoma [20–22]. Recent topical reviews elegantly highlight implications of mitochondrial dynamics on the distinct metabolism of tumor cells and stem cells [23–25]. Thus, manipulating mitochondrial function, in particular mitochondrial dynamics has emerged as an important therapeutic strategy in particular for cancer treatment, which requires new tools for drug discovery and development.

Here we show the development of a cell-based, sandwich enzyme-linked immunosorbent assay (ELISA) that enables monitoring of DRP1^Ser616^ phosphorylation in a high-throughput screening (HTS) format. The sensitive, luminescence-based assay is applicable to assess innate immune cells such as human monocyte-derived macrophages (MDMs) and monocyte-derived dendritic cells (MoDCs) as well as other cell types including cancer cell lines or biological samples. By screening of focused small molecule compound libraries we identified potent DRP1 modulators. We characterized the effects of the WNT-signaling compound ICG-001 that we found inhibits DRP1 activity in monocytes and a panel of colorectal cancer cell lines. ICG-001 activates an early ER stress pathway including activating transcription factor 4 (*ATF4)*, DNA damage inducible transcript 3 (*DDIT3*), and tribbles pseudokinase 3 (*TRIB3*) in sensitive as opposed to resistant colorectal cancer cell lines. We postulate that monitoring of early gene expression of a set of ER stress response genes (or proteins) in response to ICG-001 may be predictive of efficacy of ICG-001 in anti-cancer regimens.

## RESULTS

### Development of a cell-based sandwich ELISA to detect DRP1 activation

**Figure 1:**
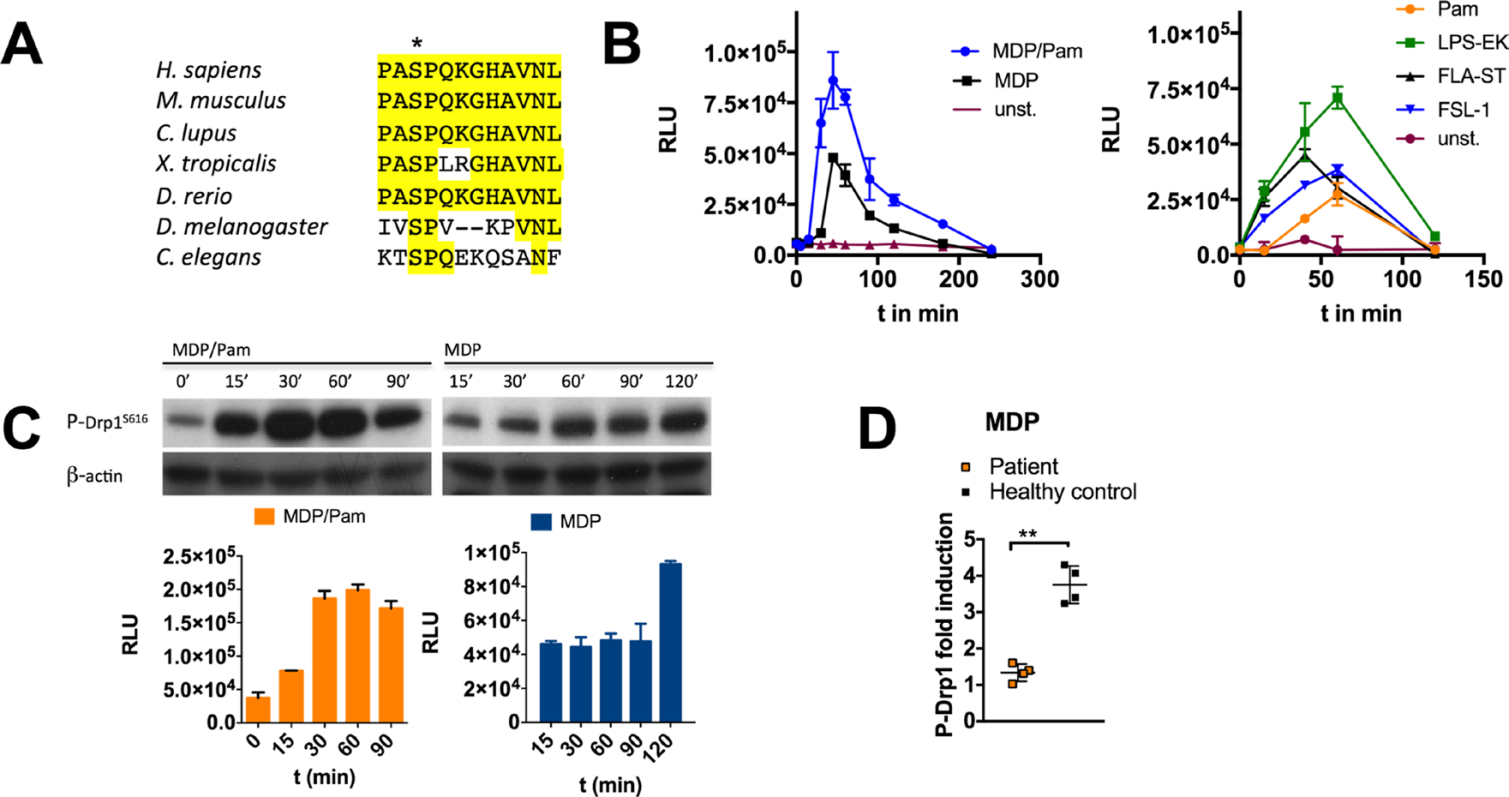
Development of a specific and sensitive sandwich-ELISA for quantification of DRP1^Ser616^ phosphorylation (**A**) DRP1^Ser616^ activating phosphorylation site is conserved among invertebrates and mammals. Sequence alignment of amino acid sequences of human DRP1 and other species as indicated. (**B**) Monitoring of P-DRP1^Ser616^ by sandwich-ELISA. MDMs were stimulated with L18-MDP (200 ng/ml), Pam (1 µg/ml), LPS-EK (50 ng/ml), FLG-ST (50 ng/ml), or FSL-1 (10 ng/ml) at indicated time points. Luminescence is measured as relative light unit (RLU). (**C**) PDRP1^Ser616^ detection by western blotting matches quantification results measured by ELISA in RAW 264.7 cell lysates. Representative western blot to detect PDRP1^Ser616^ after stimulation with MDP/Pam or MDP at indicated time points. B-actin was used as loading control. Error bars in *B* and C represent mean ± SD (*n* = 3 independent experiments) (**D**) MDMs or MoDCs isolated from blood samples of NOD2 frameshift patients or healthy donors were treated on day 5 with L18-MDP (200 ng/ml) for 1 h. P-DRP1^Ser616^ was detected by sandwich-ELISA seeding 100.000 cells / 96-well. Error bars represent mean ± SD of four biological replicates (^*^P < 0.05, ^**^P < 0.01).

DRP1 possesses the highly conserved residue serine 616 that becomes phosphorylated following a variety of stimuli (Figure 1A). Phosphorylation of this evolutionary conserved residue induces cytosolic DRP1 to translocate to mitochondria and promotes mitochondrial fission [26]. Quantitative phosphoproteomics and subsequent validation experiments including western blot analysis and confocal microscopy revealed that DRP1^Ser616^ is transiently phosphorylated upon stimulation of the pattern recognition receptors (PRRs) nucleotide-binding oligomerization domain containing 2 (NOD2) and toll-like receptor 2 (TLR2), which are key factors of the innate immune system (Supplementary Figure 1). To gain a deeper understanding of DRP1-mediated signal transduction pathways, including upstream signaling events, we developed a cell-based ELISA to identify DRP1^Ser616^ modulators. By using a mouse monoclonal capture antibody to bind DRP1 and a rabbit monoclonal detection antibody, which specifically recognizes the serine 616 phosphorylation site, we established an ELISA with high specificity. We tested several parameters which are critical to assay performance, most importantly the choice and order of the antibodies. By optimizing additional components such as cell seeding density, the composition of cell lysis buffer and blocking buffer, microtiter plates, and substrate we designed a cell-based screening assay in a 96-well format. Furthermore, a luminescence-based readout provides high sensitivity with a great dynamic range (Supplementary Figure 2). The assay is adaptable to high throughput screening (HTS) protocols and to other platforms such as the Meso Scale Discovery technology (MSD) that is based on a combination of patterned arrays and electrochemiluminescence detection (data not shown). With the developed assay we were able to assess DRP1^Ser616^ phosphorylation status in myeloid cells including MoDCS and MDMs as well as in cell lines. Stimulation experiments revealed transient phosphorylation of DRP1^Ser616^ upon activation through the PRRs NOD2 and TLR2, 4, 5, and 6 by triggering with their respective ligands muramyl dipeptide (MDP), Pam_3_CysK_4_ (Pam), lipopolysaccharide (LPS), flagellin (FLA-ST) and synthetic diacylated lipopeptide Pam2CGDPKHPKSF (FSL-1) (Figure 1B). Furthermore, we validated ELISA data by western blot results (Figure 1C). The NOD2 receptor is the strongest candidate of known susceptibility genes that are associated with Crohn’s disease (CD), a common chronic inflammatory bowel disorder. Given the pivotal function of NOD2 in autophagy induction and immune regulation that are impaired in CD, we investigated DRP1^Ser616^ phosphorylation status in blood samples from CD patients [27]. MDMs or MoDCs were derived from peripheral blood monocytes from CD patients with a frame-shift in *NOD2*. Our data show that patient cells were deficient for activation of DRP1 when stimulated with MDP (Figure 1D). Therefore, DRP1 might serve as biomarker to be used to identify novel therapeutic targets to restore NOD2 signaling defects in CD and to stratify patients in this disease.

### Screening of focused small-molecule libraries

**Figure 2:**
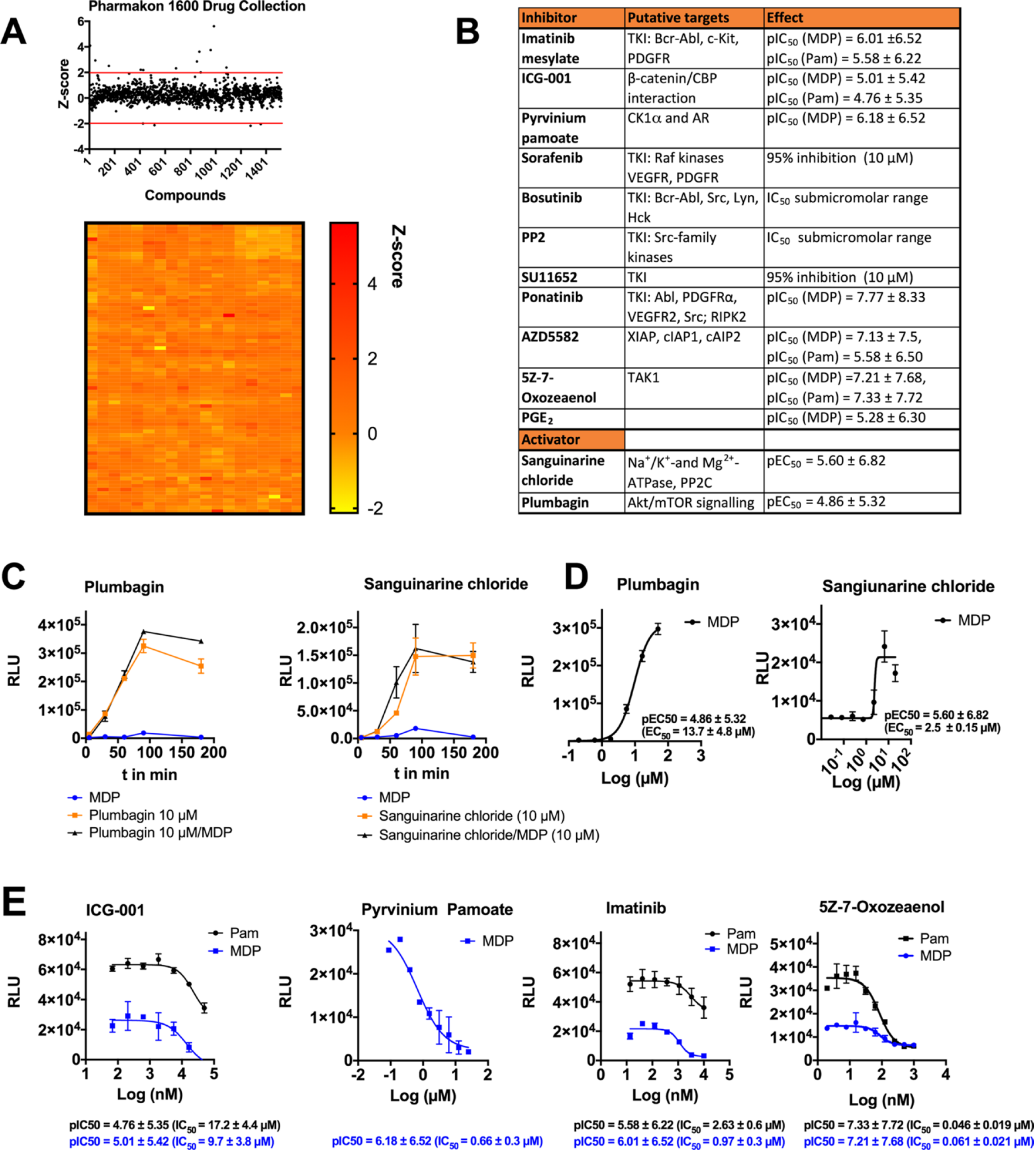
Screening of small molecule libraries identifies novel modulators of DRP1 activity (**A**) Scatterplot and heat map showing activities of 1520 compounds of the Pharmakon 1600 Drug collection. MDMs were treated with test compound (10 µM) for 1 h, following stimulation with L18-MDP/ Pam (200 ng/ml and 1 µg/ml, respectively) for 1 h. Subsequently cell lysates were analyzed by sandwich-ELISA. Drug screening was performed with technical duplicates. (**B**) List of selected identified modulators including reported targets as well as pIC_50_ or pEC_50_ (negative logarithm of the half-maximal inhibitory or effective concentration) values. (**C**) MDMs were pretreated with 10 µM plumbagin or 10 µM sanguinarine chloride for 30 min and then stimulated with L18-MDP (200 ng/ml) for indicated time points. (**D**) PEC_50_ values calculated of dose response curves of plumbagin and sanguinarine chloride. (**E**) PIC_50_ values calculated of dose response curves for ICG-001, pyrvinium pamoate, imatinib, and 5Z-7-oxozeaenol. MDMs were pretreated with various activator or inhibitor concentrations for 30 min and stimulated with L18-MDP (200 ng/ml) or 1 µg/ml Pam for 1 h. Each compound was serially diluted in DMSO and tested in a concentration range between 5100 nM and 10-50 µM, depending on pIC_50_/pEC_50_ values. Error bars in C–E represent mean ± SD of technical triplicates from at least two independent experiments. PIC_50_/pEC_50_ values were calculated using GraphPad Prism software.

To discover small-molecule modulators of DRP1^Ser616^ we performed screening experiments using several focused small compound libraries including MicroSource PHARMAKON 1600 drug collection; SCREEN-WELL WNT Pathway library; SCREEN-WELL Autophagy library; and Prostaglandin Screening libraries I, II, and III, comprising over 2050 compounds. We initially designed and executed a proof of concept screen using the MicroSource Pharmakon library of 1600 known drugs that have reached clinical evaluation and demonstrated biological activity against known targets. Also, because all of the drugs in the library are approved by the Food and Drug Administration (FDA), repositioning of drugs could be used to identify potential new therapeutic uses. The screen yielded an average Pearson correlation of 0.85 and an average Z-factor of 0.66 demonstrating a good dynamic range between negative and positive controls with robust reproducibility between replicates. We calculated a Zscore for all library plates and set a cut-off of > ± 2 SD for positive hits as either activators or inhibitors of DRP1^Ser616^. We identified 13 potential activating compounds and 4 inhibitors (Figure 2A, 2B). Out of these pyrvinium pamoate and sanguinarine chloride were evaluated and characterized as potential inhibitor and activator, respectively (Figure 2C–2E). The anthelmintic drug pyrvinium pamoate is a cytotoxic anti-cancer compound that targets mitochondrial respiration by inhibiting mitochondrial complex I [28]. Sanguinarine chloride, an alkaloid from the roots of plants of the *Papaveraceae* family is suggested to be a selective inhibitor of protein phosphatase 2 C (PP2C) and exerts anti-inflammatory effects. Additionally, we screened three target-focused small molecule libraries to interrogate effects of WNT-signaling and autophagy modulators as well as prostaglandins. Both autophagy and WNT-signaling pathways target and are regulated by mitochondrial dynamics and metabolism [29, 30]. As in target-focused libraries more active compounds shift the Z-score so that potential hit compounds may be missed we defined for these libraries a Z-score cut-off of > ± 1.5 SD, resulting in 26 identified inhibitors and 19 potential activators. The average calculated Z-factor was 0.61 (Supplementary Tables 1, 2, Supplementary Figure 3A–3C). We reacquired a large number of identified active compounds, determined dose response curves, and calculated corresponding pIC_50_/pEC_50_ values (negative logarithm of IC_50_/EC_50_; Figure 2B–2E). We observed that IC_50_ values of imatinib mesylate, pyrvinium pamoate, ICG-001, and the transforming growth factor-beta-activated kinase 1 (TAK1) inhibitor 5Z7Oxozeaenol coincide to reported IC_50_ values described for their identified targets implicating a specific target effect of these compounds (Figure 2E). Imatinib mesylate, which is an effective drug for the treatment of chronic myeloid leukemia by targeting the dysregulated tyrosine kinase BCR-ABL, inhibits the phosphorylation of DRP1^Ser616^ with an IC_50_ of 0.97 ± 0.3 µM. At this concentration imatinib mesylate, also known as Gleevec, hampers the activity of only a few tyrosine kinases including Abelson tyrosine-protein kinase 1/2 (ABL1/2), KIT proto-oncogene receptor tyrosine kinase (KIT), platelet derived growth factor receptor beta (PDGFR), and discoidin domain receptor tyrosine kinase 1/2 (DDR1/2) [31]. In addition, other tyrosine kinase inhibitors such as bosutinib, ponatinib, PP2, sorafenib, and SU11652 potently blocked DRP1^Ser616^ phosphorylation (Figure 2B). Plumbagin, a naphthoquinone present in the roots of the medicinal herb *Plumbago zeylinica,* manipulates a number of fundamental cellular processes [32]. Interestingly, it strongly elicits the phosphorylation of DRP1^Ser616^ in a concentration- and time-dependent manner (Figure 2C, 2D). TAK1 is a hub kinase that is key in innate and adaptive immunity as well as carcinogensis and mediates highly context-specific responses in different cell types [33]. 5Z-7-oxozeaenol inhibits DRP1 phosphorylation with IC_50_ values in the subnanomolar range shedding a new light on a regulatory function of TAK1 for mitochondrial dynamics (Figure 2E).

### Exploration of the effect of ICG-001 on DRP1 activation

**Figure 3:**
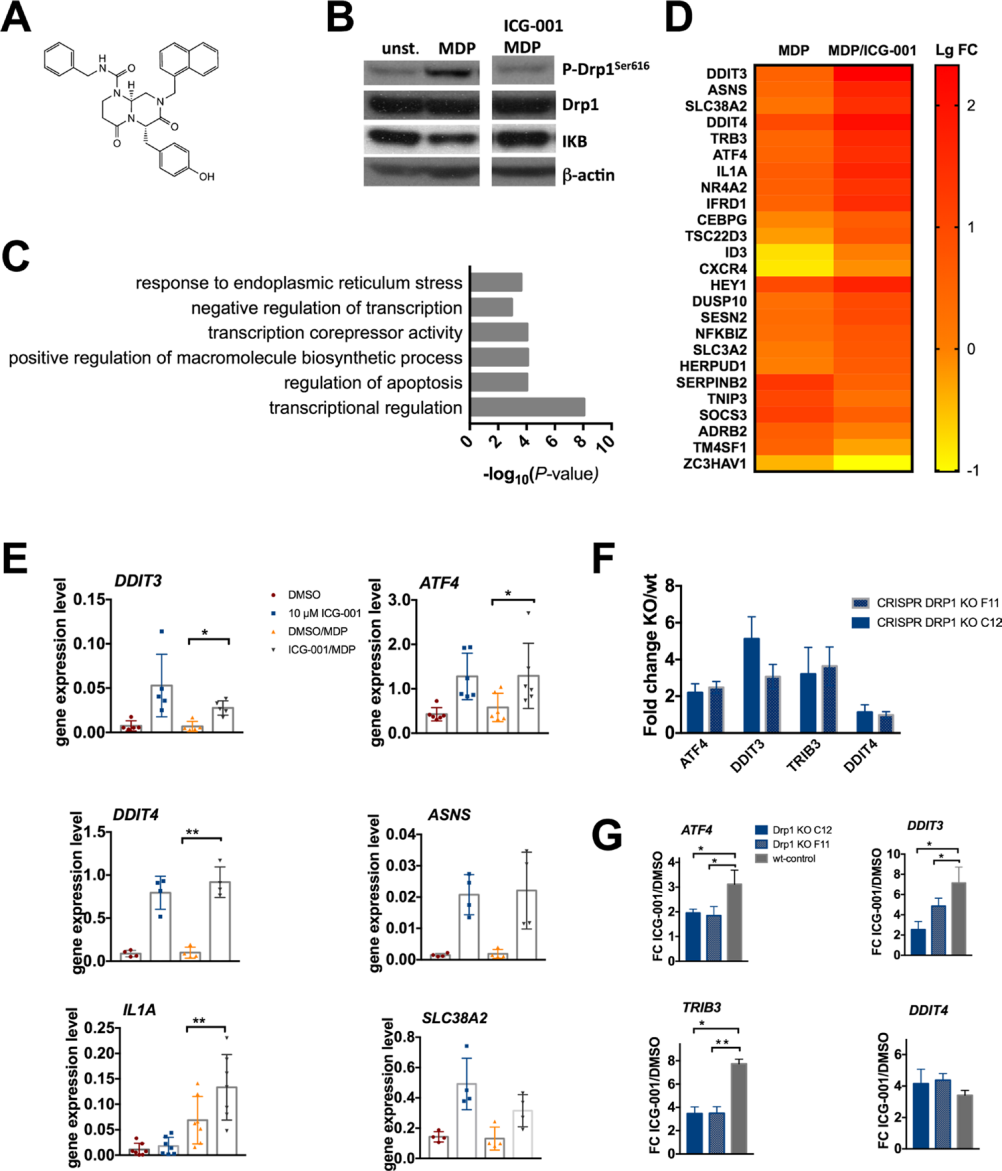
ICG-001 modulates ER stress response gene expression (**A**) Chemical structure of ICG-001. (**B**) ICG001 impairs NOD2 mediated phosphorylation in MDMs. MDMs were pretreated with 10 µM ICG-001 or vehicle control for 30 min followed by stimulation with L18-MDP (200 ng/ml) for 1 h. Cell lysates were analyzed with antibodies against P-DRP1^S616^, DRP1 and IkB. Results are shown as representative blots of two independent experiments. (**C**) Top functional categories of genes significantly enriched in response to ICG-001. Gene ontology enrichment analysis was performed on significantly upregulated genes with log_2_ FC > 0.7 (*P* < 0.01) using DAVID (**D**) Heat map showing log_2_ fold change (log FC) from MDMs treated with vehicle control or ICG-001 (as determined by microarray analysis). RNA was extracted from three biological samples after pretreatment with 10 µM ICG-001 or DMSO for 30 min and subsequent stimulation with MDP for 2 hours. (**E**) qPCR data confirm ICG-001 mediated gene expression modulation of *ATF4*, *DDIT3*, *DDIT4*, *SLC38A2*, *IL1A*, and *ASNS* in MDM, *n* = 4–6 biological samples. Error bars represent mean ± SD, ^*^P < 0.05, ^**^P < 0.001) (F) Gene expression of *ATF4*, *DDIT3*, *TRIB3*, and *DDIT4* in trans-differentiated BLaER1 DRP1 KO cell lines C12 and F11 calculated as fold change in relation to wt control. (G) Activation of ER stress response genes *ATF4*, *DDIT3*, *TRIB3*, but not *DDIT4* in DRP1 KO cells is diminished. BLaER1 DRP1 KO cell lines C12 and F11 and a corresponding wt-control cell line were treated with 10 µM ICG-001 or vehicle control for 2.5 h. *ATF4*, *DDIT3*, *TRIB3*, *DDIT4* gene expression was assed by qPCR. Error bars show mean ± SD (*n* = 3 independent experiments).

Canonical WNT/β-catenin is implicated in the development of cancer, and affects maintenance of cancer stem cells and metastasis. It has been linked to mitochondrial fission only previously [34]. The small molecule ICG-001 (Figure 3A) specifically competes with β-catenin for binding to CREB-binding protein (CBP) to inhibit WNT-signaling driven transcription, but binding of β-catenin to the highly homologous p300 is not affected [35]. ICG001 inhibits NOD2-mediated phosphorylation of DRP1^Ser616^ with an IC_50_ of 9.7 ± 3.8 µM whereas it abrogates TLR2-mediated signaling with an IC_50_ of 17.2 ± 4.4 µM. It is well established that stimulation of the membrane TLRs results in different effects, particularly in terms of signaling strength and regulation of gene expression, than stimulation of the cytosolic NOD2 receptor [36–38]. Consistent with this it is reasonable that determined IC_50_ values vary between differing PRRs. We reasoned if and how the effects of ICG-001 on β-catenin dynamics and WNT-driven transcriptional regulation are intertwined with its effect on DRP1. To verify our screening data we performed western blot analysis, thereby confirming that ICG-001 abrogates NOD2-triggered DRP1 phosphorylation in macrophages (Figure 3B). To explore how ICG-001 might act we undertook gene expression profiling in ICG-001treated MDMs. Microarray data and gene ontology enrichment analysis revealed differential expression of genes that are involved in transcriptional regulation, apoptosis and ER stress response pathways (Figure 3C). Genes that were differentially regulated with a log_2_ FC ± 1.2 are visualized in a heat map (Figure 3D). Of note, many genes encode transcription factors such as DDIT3, ATF4, and TSC22D3. TM4SF1 and SerpinB2 were previously implicated in cancer development particularly in cancer stem cell biology. ICG-001-upregulated transcripts *ATF4*, *DDIT3*, *TRIB3*, *SLC38A2*, *DDIT4*, and *ASNS* are suggested to be key players in ER stress induced Unfolded Protein Response (UPR) / Integrated Stress Response (ISR) [39]. We confirmed by real-time quantitative PCR (qPCR) increased expression of these genes in ICG-001 treated macrophages (Figure 3E). To elucidate a possible interplay between the activation of ER stress genes and ICG-001-mediated inhibition of DRP1 we investigated the effect of ICG001 in DRP1 clustered regularly interspaced short palindromic repeats (CRISPR) knockout (KO) cells and wild-type control cells. Here we utilized BLaER1 cells, immortalized human B cells that develop a myeloid gene expression pattern with a morphology resembling monocytes or macrophages upon trans-differentiation [40, 41]. Our data show that compared to wild-type control cells basal level of ER stress induced *ATF4*/*DDIT3*/*TRIB3* gene expression is elevated in DRP1 KO cell lines (Figure 3F). Moreover, DRP1 KO cells are less responsive to ICG-001 treatment (Figure 3G). Although Drp1 is presumably not the direct target of ICG-001, it appears to be involved in activating the ER stress genes *ATF4*, *DDIT3*, and *TRIB3,* but not *DDIT4*. Taken together, we found as well activation of ER stress triggered by ICG-001 is not restricted to primary cells but can also be observed in cell lines.

### ICG-001 inhibits DRP1 ^Ser616^ phosphorylation in colorectal cancer cells

**Figure 4:**
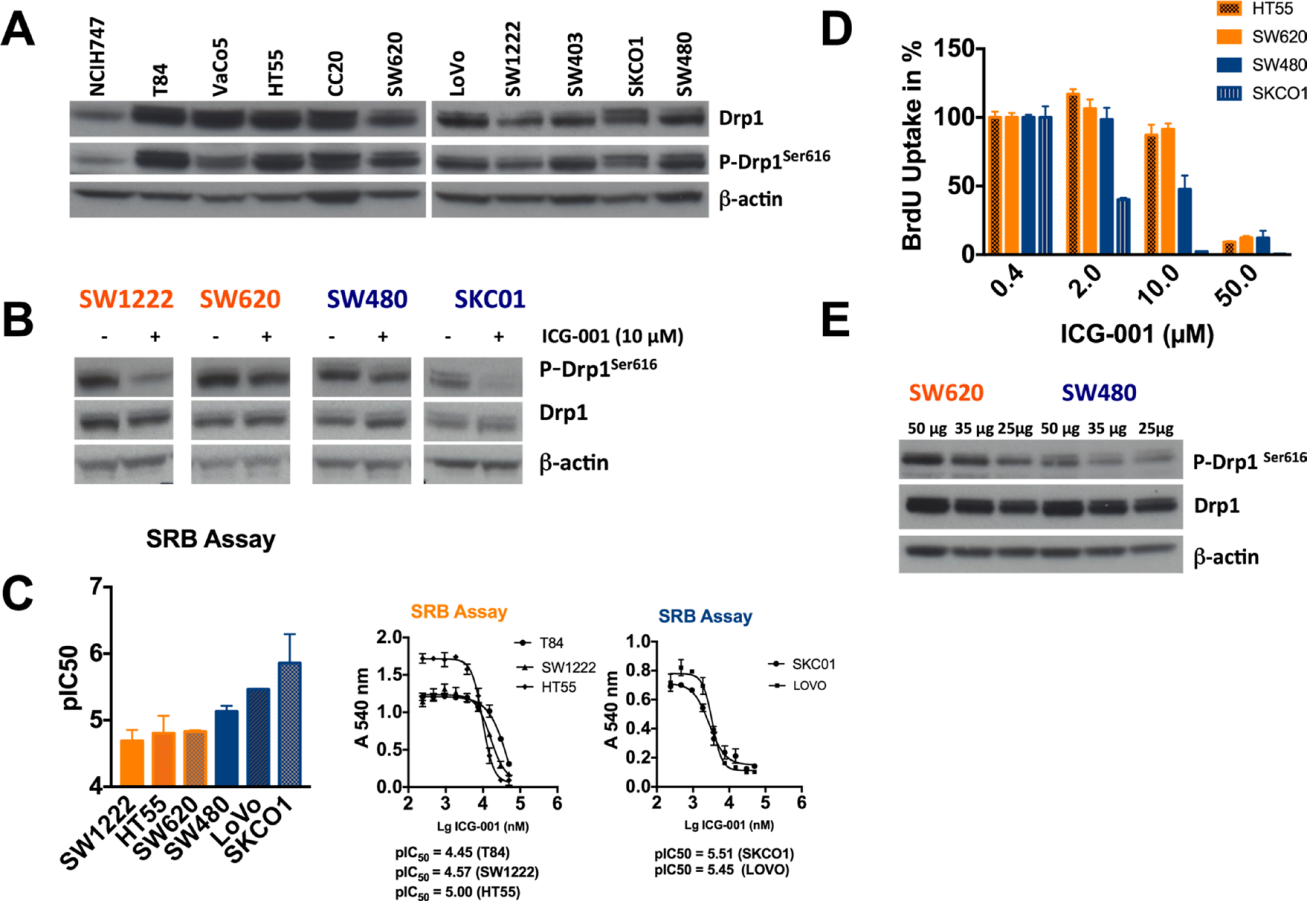
Effect of ICG-001 on a panel of colorectal cancer cell lines (**A**) Western blot analysis of P-DRP1^Ser616^ and DRP1 in a panel of 11 CRC cell lines. All cell lines carry a KRAS mutation (HT55, SW1222, and CC20 are KRAS wild type controls, (**B**) ICG-001 inhibits phosphorylation of DRP1^Ser616^ in various colorectal cancer cell lines. 1x10^6^ of SW1222, SW620, SW480 or SKCO1 cells were seeded in 6-well plates and following overnight incubation cells were treated with vehicle control or 10 µM ICG-001 for 6 h. Subsequently, western blot analysis of P-DRP1^Ser616^, DRP1, and b-actin as loading control was performed. (**C**) ICG-001 inhibits human colorectal cancer cell growth. T84, SW1222, HT55, SW620, SW480, LoVo, and SKCO1 cells were treated with a serial dilution of ICG-001 (240 nM to 50 µM) for 72-96h. Cell growth was determined by means of SRB assay. pIC_50_ values were calculated using GraphPad Prism software. (**D**) HT55, SW620, SW480 and SKCO1 cells were treated with indicated concentrations of ICG-001, and BrdU incorporation was assessed using the BrdU proliferation assay (Roche). Error bars in C and D represent mean ± SD (*n* = 3 independent experiments). (**E**) Genetically identical cell lines SW620 and SW480 were analyzed by western blot with antibodies against PDRP1^Ser616^, DRP1, and b-actin as loading control. Western blots in A, B and E are representative of at least two independent experiments.

Mounting evidence underpins a function of DRP1^Ser616^ phosphorylation in cancer progression and tumor growth. Recent reports show that RAS/ERK signaling driven activation of DRP1 contributes to cellular transformation in human melanoma and pancreatic cancer cell lines. Loss of DRP1 prevents RAS-associated mitochondrial dysfunction and results in reduced tumor growth [12, 13] and *vice versa* DRP1^Ser616^ phosphorylation phenocopies transformation-induced effects. Thus, mitochondrial fission inhibitors might provide efficient tools that interfere with MAPK-driven cancers. Hence we assessed the effect of ICG-001 on phosphorylated DRP1 in selected colorectal cancer (CRC) cell lines. First we examined endogenous levels of DRP1^Ser616^ in a panel of CRC cell lines carrying oncogenic KRAS mutations such as NCIH747, T84, VaCo5, SW620, LoVo, SW403, SKCO1, and SW480 including the wild-type controls HT55, CC20, and SW1222 (Figure 4A). We observed that DRP1^Ser616^ phosphorylation was diminished in both cancer cell lines carrying KRAS mutations and wild-type control cell lines such as SW1222 upon treatment with 10 µM ICG-001 for 6 h (Figure 4B). This suggests ICG-001 might not be employed in oncogenic RAS-driven, ERK-mediated DRP1 activating signaling but acts via another molecular mechanism. Notably, we further noticed that ERK1/2 phosphorylation is unaltered in ICG-001-treated cells (Supplementary Figure 4D). In contrast we observed in sensitive cell lines that p-AKT^Ser473^ was strongly induced after 6h, though phosphorylation of its substrate GSK-3β was unaltered (Supplementary Figure 4C, 4Ε).

To further analyze effects of ICG-001 efficacy we treated CRC cell lines with 10 µM ICG-001 for 7296 h, corresponding to three doubling times, and performed the Sulforhodamine B (SRB) assay and CellTiter-Glo assay to assess cell viability. Data analysis showed that ICG-001 inhibited cell viability of several cell lines, whereas SW1222 and T84 were resistant to ICG-001 treatment. PIC_50_ values (negative logarithm of IC_50_) for sensitive cell lines such as SKCO1 and LoVo were 5.55 and 5.47 (SRB) and 5.57 and 5.26 (CellTiter-Glo), respectively (Figure 4C, Supplementary Figure 4A, 4B). We found that growth inhibition was associated with decreased proliferation, measured by Bromodeoxyuridine (BrdU) incorporation. Data revealed that treatment with 2 or 10 µM ICG-001 resulted in inhibition of BrdU incorporation in sensitive cell lines such as SKCO1 and LoVo (Figure 4D). In summary, based on our data, we could not correlate the extent of DRP1^Ser616^ phosphorylation inhibition with sensitivity of the cell lines following ICG-001 treatment. However, we observed in SW620 cells, the metastatic variant of the primary colon tumor SW480, significantly higher phosphorylation of DRP1^Ser616^ (Figure 4E). This is in line with a suggested role of DRP1^Ser616^ phosphorylation in cancer cell metastasis as depicted in breast cancer [20].

### Upregulation of ER stress response genes correlates with ICG-001 sensitivity in colorectal cancer cells

**Figure 5:**
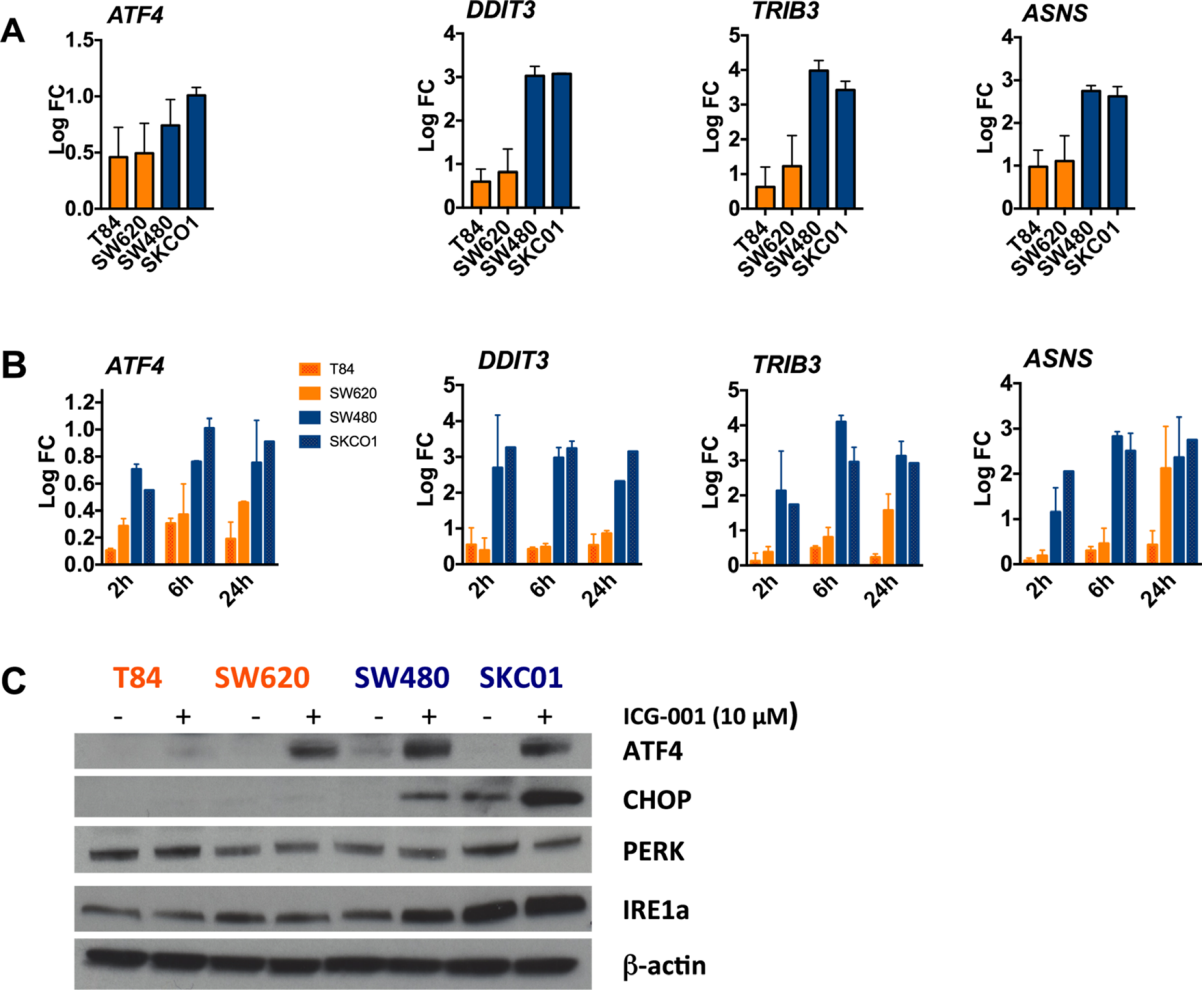
Activation of ER stress response genes in ICG-001-sensitive CRC cell lines (**A**) ER stress response genes *ATF4*, *DDIT3*, *TRIB3* and *ASNS* are activated in sensitive cells but not in resistant cell lines. Cells were treated with 10 µM ICG-001 for 6 hours. Gene expression was assessed by qPCR. Data are presented as log_2_ fold change (log FC), normalized to vehicle controls. Error bars show mean ± SD (*n* = 3 independent experiments). (**B**) Gene expression of *ATF4*, *DDIT3*, *TRIB3*, and *ASNS* in resistant cell lines (T84, SW620) and sensitive cell lines (SW480, SKCO1) analyzed by qPCR. Cells were treated with 10 µM ICG-001 for 2, 6 and 24 h. Expression data are presented as log_2_ FC, normalized to vehicle controls. Time course results are expressed as mean ± SD of two independent experiments with triplicates. (**C**) Indicated cell lines were treated with 10 µM ICG-001 for 6 h. Whole cell lysates were analyzed by western blot analysis with antibodies against ATF4, CHOP, PERK, IRE1a, and b-actin as loading control. Representative western blot of two independent experiments are shown.

Gene expression profiling of macrophages treated with ICG-001 displayed significant transcriptional activation of a series of ER stress response genes such as *ATF4*, *DDIT3*, *TRIB3*, *DDIT4*, *SLC38A2* and *ASNS* (Figure 3D, 3E). We further interrogated these findings by qPCR in a panel of CRC cell lines treated with 10 µM ICG-001 for 6 h. We observed a distinct correlation between induction of *ATF4*, *DDIT3*, *TRIB3*, and *ASNS* and sensitivity in response to ICG-001. In sensitive cell lines including SKCO1 and LoVo *ATF4*, *DDIT3*, *TRIB3*, and *ASNS* mRNA levels were clearly increased whereas resistant cell lines (T84, SW1222 and HT55) exhibited only minor or no transcriptional activation (Supplementary Figures 5A, 4E). We found a similar effect already after 2 h as demonstrated in time course experiments. Increased gene expression of *ATF4*, *DDIT3*, *TRIB3*, and *ASNS* persisted after 24 h of treatment (Figure 5B). We then assessed if the induction of ER stress response genes is accompanied by translational changes of the corresponding proteins. Western blot analysis revealed increased abundance of ATF4 and C/EBP homologous protein (CHOP), that is encoded by *DDIT3*, in ICG-001-sensitive cell lines such as SW480 and SKCO1, but not in in resistant cell lines after 6 h (Figure 5C). The abundance of other ER stress proteins such as protein disulfide isomerase (PDI), inositol-requiring kinase 1 (IRE1a), and PKR-related ER kinase (PERK) was unchanged in resistant and sensitive cell lines (Supplementary Figures 5C, 4E). Thus ICG-001 induces an early and specific ER stress response pathway in cell lines susceptible to its anti-proliferative activity.

## DISCUSSION

Due to the key role of mitochondria in health and disease it is of great interest to define modulators of mitochondrial dynamics that correct functional imbalances including perturbed DRP1 activity. Here we show the development of a cell-based screening assay that enables the detection of the activating serine 616 residue of the mitochondrial fission protein DRP1. Taken together, our screening experiments revealed a number of new and supposed mitochondrial modulators. Identified DRP1 activators such as A23187, ionomycin, tunicamycin and thapsigargin that cause an increase of intracellular Ca^2+^ and/or trigger ER stress are known to be implicated in the regulation of mitochondrial dynamics (Supplementary Table 2). Further, we identified potent inhibitors that target mitochondria such as [42] the anthelmintic drugs niclosamide and a well-characterized inhibitor of mitochondrial complex I, rotenone (Supplementary Table 1). Recently it was shown that canonical WNT/β-catenin signaling regulates mitochondrial metabolism and dynamics causing modified oncogenic signaling in melanoma cells [34]. Noncanonical WNT-5A induced phosphorylation of DRP1^Ser616^ in neurons, is concomitant with increased mitochondrial fission [43]. Hence the WNT-signaling compound ICG-001 captured our interest as a DRP1^Ser616^ phosphorylation inhibitor. Gene expression profiling in ICG001-treated macrophages revealed activation of ER stress response genes such as *ATF4*, *DDIT3*, *TRIB3*, and *ASNS*. Intriguingly, ATF4 has recently been identified as key regulator of mitochondrial stress response [44]. Beyond its described effect on WNT-mediated transcriptional regulation ICG-001 induces an ER stress response pathway that we show is linked to ICG001 efficacy in a panel of CRC cell lines. Moreover, the compound fails to induce upregulation of an *ATF4/DDIT3/TRIB3* axis in ICG-001-resistant cells. Our findings uncover a novel MOA of ICG-001 that is linked to its anti-proliferative effect. Of note, recent data suggest ICG-001 inhibits pediatric glioma tumorigenicity and multiple myeloma growth in a Wnt-independent manner [45, 46].

In general, it is still obscure whether and under which circumstances ER stress response signaling diminishes or promotes cancer cell survival or tumor growth. Many studies show that activation of the ER stress response favors tumorigenicity and metastasis under tumor microenvironment conditions characterized by glucose and amino acid deprivation and oxidative stress (hypoxia) [47]. However, unresolved or massive ER stress can lead to apoptosis though underlying molecular mechanisms are poorly understood. So far only a few studies describe drugs that employ a signaling cascade triggering cell death in cancer cells but not in untransformed cells. The anti-cancer compound HA15 induces an early ER stress response leading to apoptosis and autophagy-mediated cell death in melanoma cells. It displays strong anti-tumorigenic activity in xenograft mouse models with melanoma cells, which depends on activation of ATF4 and DDIT3, without toxicity in normal cells [48]. The anti-cancer effects of the first-in-class molecule ONC201 depend on the activation of an ATF4 signaling pathway [49–52]. Further, cannabinoid-mediated apoptosis is associated with the upregulation of ATF4, DDIT3, and TRIB3 in cannabinoid-sensitive glioma cells [53]. These and our findings may provide a basis to develop HTS protocols to screen for anti-cancer compounds by means of qPCR-based early detection of *DDIT3* and *TRIB3* (t = 6 h).

In summary, mitochondrial dysfunction plays a fundamental role in human disease. Here we describe a straightforward, robust cell-based assay for use in high throughput screening (HTS) applications to define modulators of mitochondrial dynamics and morphology. This could be utilized for genetic screens to define control mechanisms of mitochondrial dynamics in diverse cells types and also for definition of new drugs targeting these pathways.

## MATERIALS AND METHODS

### Compounds and reagents

Small molecule libraries and chemical compounds: The Pharmakon 1600 drug library (Microsource Discovery Systems) was provided by the Target Discovery Institute (TDI), Oxford University. The SCREEN-WELL^®^ Autophagy library (BML-28) and SCREEN-WELL^®^ WNT Pathway library (BML-2838) were purchased from Enzo Life Sciences. Prostaglandin Screening libraries I, II and III (cat. No. 10501, 10502, and 10503) were purchased by Cayman Chemical Company. Curcumin (08511 cat. no.), pyrvinium pamoate (cat. no. P0027), 5Z-7-Oxozeaenol (cat. no. O9890), plumbagin (cat. no. P7262), A23187 (cat. no. C7522), and thapsigargin (cat. no. T9033) were purchased from SIGMA. AZD 5582 (cat. no. 5141) and ICG-001 (cat. no. 4505) were purchased from Tocris. Imatinib mesylate (cat. no. CAY13139), mdivi (cat. no. CAY15559), bosutinib (cat. no. CAY12030), PP2 (cat. no. CAY13198), ponatinib (cat. no. CAY11494), sanguinarine chloride (cat. no. CAY16951) were received from Cambridge Bioscience. Akt/PDK1/Flt dual pathway inhibitor (cat. no. 521275) was purchased by EMD. Following Antibodies were purchased from Cell Signaling Technology: DRP1 (5391), P-DRP1^(Ser616)^ (4494) P-p44/42 MAPK (ERK1/2) ^(Thr202/Tyr204)^(4370), p44/42 (ERK1/2) (4695), P-AKT ^(Ser473)^ (4060), AKT (9272), b-actin (4967), CHOP (2895), Grp75 (3593), PERK (5683), PDI (3501), IRE1 ± (3294), Phospho-GSK-3^2^ (Ser9) (5558), GSK-3^2^ (12456), IkB-a (9242), Anti-rabbit IgG, HRP-linked Antibody (7074), Anti-mouse IgG, HRP-linked Antibody (7076).

### Sandwich ELISA

Protocol: Each well of a white 96-well microtiter plate was coated overnight at 4°C with 100 µl capturing anti-mouse DRP1 antibody. The final concentration of the DRP1 antibody diluted in phosphate buffered saline (PBS) was 0.8 ng/ml. After 3 washes with 250 µl PBST (PBS with 0.05% Tween 20), 200 µl of blocking solution (0.2% ECL™ Blocking Agent in PBST) was added and plates were incubated for 1 h at RT. After one washing step with 250 µl PBST, 100 µl sample was added and incubated for 90 min at RT. After three washing steps with 250 µl PBST, primary P-DRP1^Ser616^ antibody was added and incubated for 90 min at RT. Primary P-DRP1^Ser616^ antibody was 1:500 diluted in blocking solution. After three washing steps with 250 µl PBST, 100 µl secondary HRP-labeled antibody solution (1:500 diluted in blocking solution) was applied and incubated for 1 h. Following four final washing steps with 250 µl PBST, 100 µl ELISA Pico Chemiluminescent Substrate as detection reagent was added to each well. Luminescence signals were measured using GloMax^®^ Discover plate reader (Promega) or the Fluostar Optima (BMG Labtech) according to the instructions of the manufacturer. Using plate sealers during assay incubation steps can increase accuracy of assay performance. Sample preparation: Cell lysates were generated by adding 100 µl cell lysis buffer, complemented with protease inhibitors (PMSF, cOmplete protease inhibitor cocktail), to each 96-well. Subsequently samples were incubated on ice for 15 minutes and stored at -80°C overnight before performing the ELISA.

The following reagents and consumables were purchased for ELISA performance: 96-well microtiter plate, high binding (Costar, cat. no. 3922), Anti-mouse DRP1 (Abcam, cat. no. ab56788), Tween 20 (Promega, cat. no. H5152), ECL™ Blocking Agent (Amersham, cat. no. RPN2125), P-DRP1^Ser616^ (Cell signaling technology, cat. no. 4494), Anti-rabbit IgG, HRP-linked Antibody (Cell signaling technology, cat. no. 7074), ELISA Pico Chemiluminescent Substrate (ThermoFisher Scientific, cat. no. 37069), Cell Lysis Buffer (Cell signaling technology, cat. no. 9803), cOmplete^™^, Mini, EDTA-free Protease Inhibitor Cocktail (ROCHE, cat. no. 11836170001), PMSF (Cell signaling technology, cat. no. 8553).

### Patient consent

CD patients who are homozygous, or compound heterozygous, for NOD2 polymorphisms were identified from the Oxford IBD cohort. Informed consent was obtained from all healthy donors and patients.

### Macrophage (MDM) and dendritic cell (MoDC) isolation

Leukocyte reduction system (LRS) cone blood from healthy donors were obtained from the UK National Blood Centre, and donor samples with informed consent following local ethical guidelines granted by Milton Keynes Research Ethics Committee Ref. 07/H0603/43. We isolated monocytes from Lymphoprep (Axis-Shield) and gradient-enriched mononuclear cells by MACS CD14-positive selection (Miltenyi Biotech). To obtain macrophages we cultured the cells in RPMI 10% Fetal Bovin Serum (FBS, Sigma), 2 mM L-glutamin (Sigma), 1% penicillin streptomycin (Sigma) (complete medium) supplemented with 100ng/mL of human macrophage colony–stimulating factor (hrM-CSF, Peprotech) for 5 d at 37°C and 5% CO_2_ before use. To isolate dendritic cells we cultured CD14-enriched monocytes in complete medium supplemented with 40 ng/mL of IL-4 (Peprotech) and 40 ng/mL of human granulocyte macrophage – colony stimulation factor (hrGM-CSF, Peprotech) for 5 days before use.

### Compound library screen

MDMs were isolated from healthy donor buffy coats as described above. On day four MDMs from 3 individual donors were mixed in equal numbers and plated into 96-well plates (6 × 10^4^ cells/well). Cells were cultured in complete RPMI overnight at 37°C and 5% CO_2_. On day five medium was removed and the MicroSource Pharmakon 1600 drug library was added at a final concentration of 10 µM. Each library plate was run in technical duplicates. As positive control ponatinib (50 nM) was applied and vehicle controls (DMSO) in stimulated and unstimulated medium were added as negative controls to multiple wells on each plate as well. After 1 h pretreatment 100 µl medium including compounds and stimulant (200 ng/ml L18-MDP, 1 µg/ml Pam) was added and incubated for 1 h. Subsequently microtiter plates were washed with PBST and 100 µl lysis buffer was added. Plates were incubated on ice for at least 15 min and subsequently stored at –80°C. ELISA was performed as described above by starting with the transfer of 90 µl lysate into high-binding, white ELISA plates. Liquid handling was performed with a Janus automated workstation equipped with 96-well MDT (both Perkin Elmer, Waltham, MA). Plates were read using a Perkin Elmer Evision. For overall screen performance, Z-factor of individual plates was calculated based on positive and negative controls. Z-factors for averaged duplicates were calculated as described earlier [54].

### Confocal microscopy

2 × 10^5^ MoDCs in 250 µl RPMI (supplemented with 10% FBS) were seeded on poly-L-lysine-coated glass coverslip placed in a 24-well culture dish. Following the stimulation experiment cells were fixed with 4% paraformaldehyde in PBS, permeabilized with 0.1% Triton X-100 for 10 min, blocked with blocking solution (PBS containing 5% FCS, 5% human serum, 5% goat serum and 0.1% Triton) and incubated with P-DRP1^Ser616^ and PDI (RL90, cat. no. ab2792) antibodies overnight at 4°C, followed by incubating with fluorescence labeled antibodies (Alexa Fluor 555 goat anti-rabbit IgG, cat. no. A21428; Abberior STAR RED goat anti-mouse IgG, cat. no. 2-0002-011-2 as well as Oregon green 488 phalloidin, cat. no. O7466), mounted with DAPI-containing mounting media (NucBlue Fixed Cell Stain Ready Probes DAPI, cat. no. R37606). All images were acquired with an inverted Zeiss LSM 880 Confocal Microscope (Carl Zeiss, Jena, Germany). Images were processed with ImageJ.

### Immunoblotting

Cells were cultured in RPMI (10% FBS) in 6-well plates or Eppendorf tubes, treated, harvested and lysed. Protein concentrations of whole cell lysates were measured by bicinchoninic acid protein assay (BCA). Cell lysates (10–50 μg) were separated using NuPAGE™ 4–12% Bis-Tris Protein Gels (Invitrogen) and transferred to a polyvinylidene difluoride (PVDF) membrane (Amersham). Blocked membranes were probed overnight at 4°C with primary antibodies. Subsequently blots were incubated with corresponding anti-rabbit IgG or anti-mouse IgG secondary antibodies conjugated to horseradish peroxidase. Films were developed using enhanced chemiluminescence reaction (ECL).

### Real-time quantitative PCR (qPCR)

Total RNA was extracted from cells using RNeasy Mini Kit (Qiagen) following manufacture’s instructions. First strand cDNA was transcribed using High-Capacity RNA-to-cDNA Kit (Applied Biosystems, cat. no. 4387406) according to the manufacturer’s protocol. QPCR was performed using using TaqMan^®^ Gene Expression Assays on QuantStudio™ 7 Flex system (Applied Biosystems). Amplification was performed in 6.25 µl reactions in 384-well plates using TaqMan Fast Advanced Master Mix under recommended conditions (Applied Biosystems, cat. no. 4444557). The following TaqMan gene expression assays (Applied Biosystems) were used: ATF4 (Hs00909569_g1), DDIT3 (Hs00358796_g1), DDIT4 (Hs01111686_g1), SLC38A2 (Hs01089954_m1), ASNS (Hs04186194_m1), IL1A (Hs00174092_m1), and RPLP0 (Hs00420895_gH).

### BLaER1 and CRC cell lines

BLaER1 were cultured in RPMI Medium complemented with 10% FCS, 1% penicillin streptomycin, 1 mM sodium pyruvate (Gibco) and 2 mM L-glutamine. Cells were transdifferentiated into monocytes in medium additionally containing 10 ng/ml hrIL-3, 10 ng/ml hrM-CSF (both PeproTech), and 100 nM b-Estradiol (Sigma-Aldrich) and incubated at 37°C and 5% CO_2_ for 6 days. To perform qPCR experiments 60.000 cells were seeded into 96-well plates. Cells were stimulated in in fresh RPMI Medium 1640 supplemented with 10% FCS. We obtained all CRC cell lines T84, SW1222, SW620, HT55, SW403, Vaco5, SW480, NCI747, CC20, LoVo and SKCO1 from Walter Bodmer (WIMM, University of Oxford) Cells were cultured as described earlier in [55].

### Statistical analysis

We used GraphPad Prism v.6.05 to determine the statistical significance of differences in the means of experimental groups with unpaired or paired, two-tailed Student’s *t-*tests. Significance in the Figures is reported as ^*^P < 0.05, ^**^P < 0.01.

### Cell proliferation assay

BrdU assays were performed using chemiluminescent-based Cell Proliferation ELISA, BrdU assay kit (Roche) according to the manufacturer’s instructions. 5 × 10^3^ cells were plated per well into white 96-well plates. BrdU was added to a final concentration of 10 mM for 1 h. Peroxidase conjugated anti-BrdU antibody was added to cells for 30 to 60 min at RT and luminescence was measured after an incubation of 3 to 10 min using a GloMax^®^ Discover plate reader (Promega).

### Cell viability assays

Cancer cells (3–5 × 10^3^) were seeded into 96-well plates and incubated overnight. To determine IC_50_ values medium was removed and 200 µl fresh medium including a concentration range of 240 nM to 50 µM ICG001 was added. After three doubling times of 72–96 h of incubation, CellTiter-Glo luminescent cell viability assay (Promega,) was performed according to manufacturer’s protocols. The sulforhodamine B assay (SRB) was performed following a standard protocol [55]. Cells were fixed by the addition of ice-cold trichloroacetic acid and washed three times with water after 1 h incubation at 4°C. Cells were then stained with 100 µL SRB solution (0.04% in 1% acetic acid) for 30 min, washed three times with acetic acid (1%), and bound dye was dissolved in 200 µl Tris (10 mM, pH 9.5) before measuring the OD at 540 nm in a plate reader. The doubling times of the CRC cell lines tested are as follows: T84: 54 h; SW1222: 44 h; SW620: 26h; HT55: 47h; SW403: 38 h; SW480: 45h; VACO5: 31 h; LoVo: 27 h; SKCO1: 51 h.

### Microarray

Macrophages isolated from 3 healthy donors were treated with ICG-001 (10 µM) or vehicle control (DMSO) for 30 min, following stimulation with L18-MDP (200 ng/ml) for 2 h. Quality and integrity of extracted RNA was measured by NanoDrop and samples were analyzed on Illumina Human HT-12 chips. Microarray data were quality controlled and normalized with the R lumi package and analysed with R LIMMA package. For identification of differential expression the results were filtered using an adjusted *P* value < 0.05 [56, 57]. Microarray data have been deposited in the NCBI Gene Expression Omnibus (GEO) database and are accessible through GEO series accession number GSE102117.

## SUPPLEMENTARY MATERIALS FIGURES AND TABLES


