# Supplementary material for: ICG-001 affects DRP1 activity and ER stress correlative with its anti-proliferative effect
Source: Oncotarget. 2017 Nov 1;8(63):106764–77. doi: 10.18632/oncotarget.22264 (PMC5739772; doi:10.18632/oncotarget.22264)
Supplement: Supplementary file 1 [file oncotarget-08-106764-s001.pdf]

ICG-001 affects DRP1 activity and ER stress correlative with its anti-proliferative effect

SUPPLEMENTARY MATERIALS

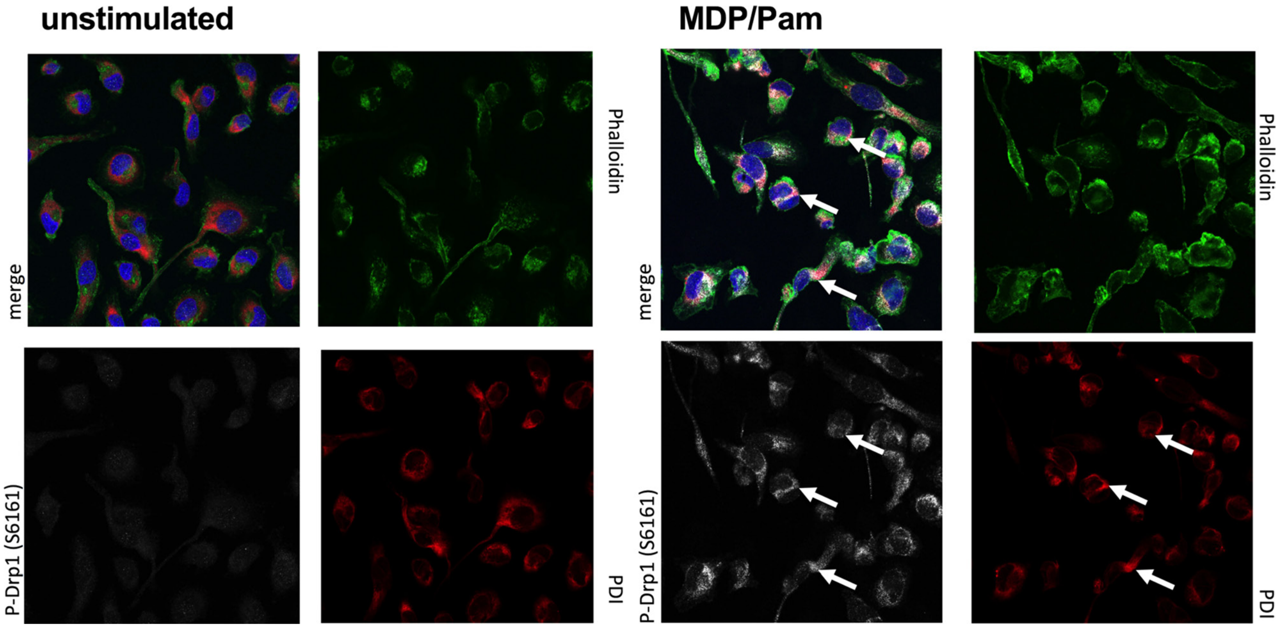

Supplementary Figure 1: DRP1 is phosphorylated upon NOD2/TLR2 receptor stimulation. Representative immunofluorescence of P DRP1Ser616 (white), Phalloidin (green) and PDI (red) in MoDCs stimulated with L-18MDP/Pam (200 ng/ml and 1 µg/ml) for 45 min and imaged. P DRP1Ser616 co-localizes with the ER-marker PDI as indicated with white arrows.

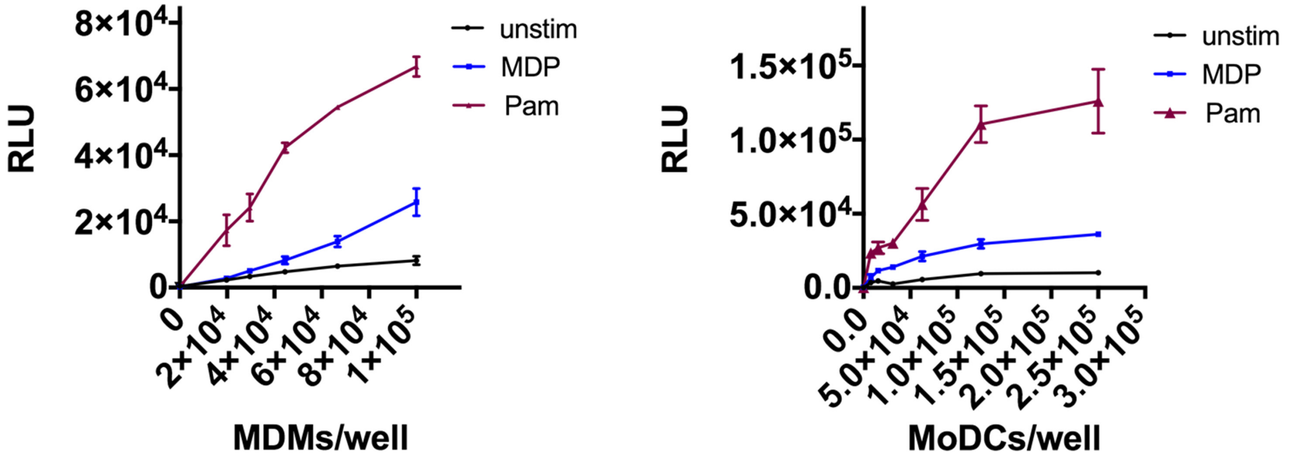

Supplementary Figure 2: P-DRP1Ser616 sandwich ELISA validation. Dose response curves: indicated amounts of MDMs were seeded into 96-well plates and following o/n incubation cells were stimulated with L18-MDP (200 ng/ml) or Pam (1 µg/µl) for 60 min. MoDCs were stimulated in Eppendorf tubes in a volume of 500 µl, harvested by centrifugation, washed with PBS and subsequently lysed by adding 100 µl lysis buffer. Cell lysates were added into 96-well Elisa plates for assay performance. Error bars represent mean ± SD (***n*** = two independent experiments).

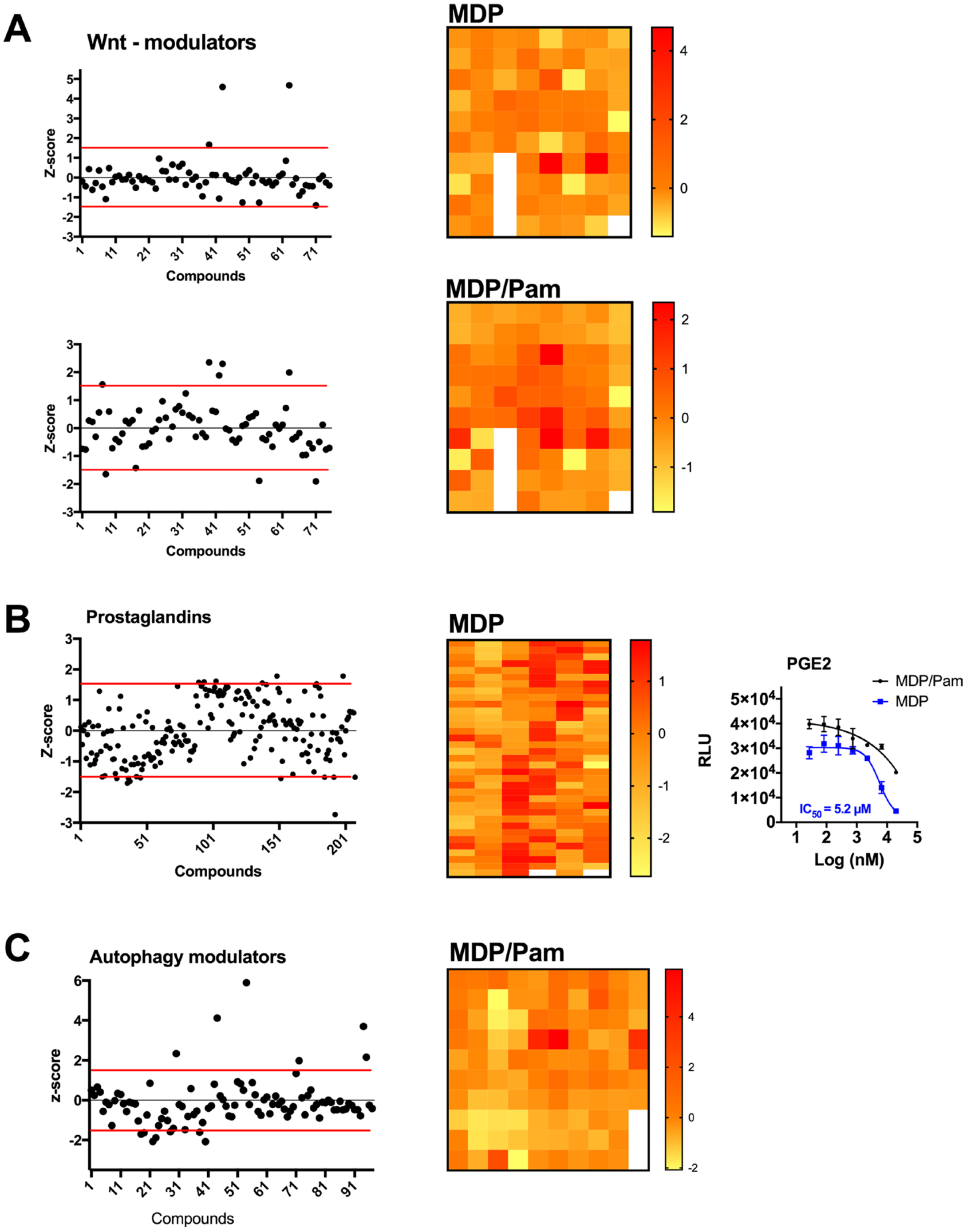

Supplementary Figure 3: Scatterplots and heat maps of screening experiments. Scatterplot and heat map showing activities of small molecules of SCREEN-WELL® WNT Pathway library (**A**), Prostaglandin Screening libraries I, II and III (**B**) and SCREEN-WELL® Autophagy library (**C**). (A–C) MDMs were treated with test compounds at a final concentration of 10 µM for 1 h, following stimulation with L18-MDP or L-18MDP/Pam (200 ng/ml and 1 µg/ml, respectively) for 1 h. Subsequently cell lysates were analyzed by sandwich ELISA. Drug screening was performed with technical duplicates in at least in two independent experiments. The respective calculated Z'-factors were A: 0.53 (MDP) and 0.56 (MDP/Pam), B: 0.59, and C: 0.67. (B) PGE2 was serially diluted in DMSO and tested in a concentration range of 100 nM – 50 µM. MDMs were pretreated with PGE2 or vehicle control for 30 min and then stimulated with L18-MDP (200 ng/ml) for 1 h.

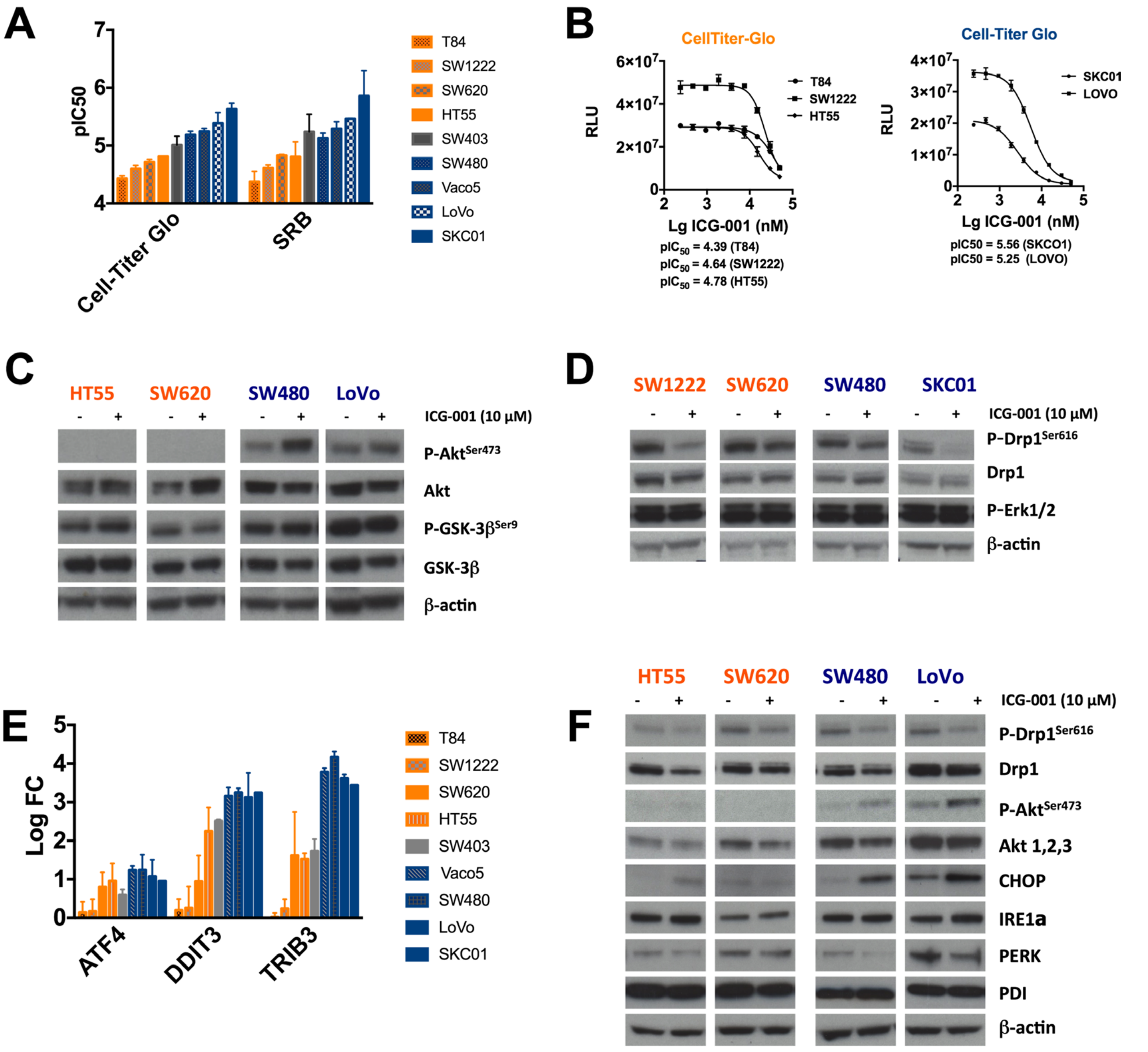

Supplementary Figure 4: ICG-001 affects cell viability and expression of ER stress response genes in a panel of colorectal cancer cells. (**A**) T84, SW1222, HT55, SW403, VaCo5, SW480, LoVo and SKCO1 cells were treated with ICG-001 (240 nM to 50 µM) for 72–96 hours, corresponding to three doubling times. Cell viability was determined by using Cell-Titer Glo and SRB assay. PIC_{50} (negative logarithm of IC_{50}) values were calculated with GraphPad Prism software. Error bars represent mean ± SD of triplicates (n = 2 independent experiments). (**B**) Dose response curves for T84, SW1222, HT55, LoVo, and SKCO1 cells were treated with a serial dilution of ICG 001 (240 nM to 50 µM) for 72–96 h. Cell growth was determined by means of Cell-Titer Glo. Error bars represent mean ± SD of triplicates (n = 2 independent experiments). PIC_{50} values were calculated using GraphPad Prism software. (**C**) Indicated cell lines were treated with 10 µM ICG-001 for 6 h. Whole cell lysates were analyzed by western blot with P-AKTSer473, AKT, P-GSK-3β^{Ser9}, GSK-3β and β-actin antibodies. (**D**) Indicated cell lines were treated with 10 µM ICG 001 for 6 h. Whole cell lysates were analyzed by western blot analysis with P-DRP1Ser616, DRP1, P-Erk1/2, and β-actin antibodies. (**E**) Gene expression of ATF4, DDIT3, and TRIB3 in indicated cell lines analyzed by qPCR. Cells were treated with 10 µM ICG-001 for 6 h. Expression data are presented as log_2 FC, normalized to vehicle controls. Error bars show mean ± SD of technical triplicates, n = 2. (**F**) Indicated cell lines were treated with 10 µM ICG-001 for 6h. Whole cell lysates were analyzed by western blot analysis with antibodies against DRP1, DRP1^{Ser616}, CHOP, P-AKT^{Ser473}, AKT, PERK, IRE1, PDI and β-actin. Western blot data in C, D and F represent at least two independent experiments.

Supplementary Table 1: Selective compounds that were identified as potential inhibitors of mitochondrial fission

Inhibitor	Putative targets	References
**Imatinib mesylate**	Antineoplastic agent that inhibits Bcr-Abl protein tyrosine kinase; specific inhibition of PDGFR and c-KIT	**Bantscheff M**, Eberhard D, Abraham Y, Bastuck S, Boesche M, Hobson S, Mathieson T, Perrin J, Raida M, Rau M, Reader V, Sweetman G, Bauer A, Bouwmeester T, Hopf C, et al. Quantitative chemical proteomics reveals mechanisms of action of clinical ABL kinase inhibitors. Nat Biotechnol. 2007; 25:10351044.
**ICG-001**	Selectively inhibits β-catenin/CBP interaction, but no effect on β-catenin/p300 interaction	**Emami KH**, Nguyen C, Ma H, Kim DH, Jeong KW, Eguchi M, Moon RT, Teo JL, Oh SW, Kim HY, Moon SH, Ha JR, Kahn M. A small molecule inhibitor of beta-catenin/CREB-binding protein transcription. PNAS. 2004; 101:12682–12687.
**Pyrvinium pamoate**	Targets Wnt-signaling and mitochondrial respiration	**Ishii I**, Harada Y, and Kasahara T. Reprofiling a classical anthelmintic, pyrvinium pamoate, as an anti-cancer drug targeting mitochondrial respiration. Front Oncol. 2012; 2:137. **Lamb R**, Ozsvari B, Lisanti CL, Tanowitz HB, Howell A, Martinez-Outschoorn UE, Sotgia F, Lisanti MP. Antibiotics that target mitochondria effectively eradicate cancer stem cells, across multiple tumor types: treating cancer like an infectious disease. Oncotarget. 2015; 6:4569–4584.
**Sorafenib**	Blocks RAF kinase, a component of the RAF/MEK/ERK signaling; inhibits the VEGFR-2/PDGFRβ signaling	**Tesori V**, Piscaglia AC, Samengo D, Barba M, Bernardini C, Scatena R, Pontoglio A, Castellini L, Spelbrink JN, Maulucci G, Puglisi MA, Pani G, Gasbarrini A. The multikinase inhibitor Sorafenib enhances glycolysis and synergizes with glycolysis blockade for cancer cell killing. Sci. Rep. 2015; 5:9149.
**Bosutinib**	Dual kinase inhibitor that targets both Abl and Src kinases with potential antineoplastic activity	**Bantscheff M**, Eberhard D, Abraham Y, Bastuck S, Boesche M, Hobson S, Mathieson T, Perrin J, Raida M, Rau M, Reader V, Sweetman G, Bauer A, Bouwmeester T, Hopf C, et al. Quantitative chemical proteomics reveals mechanisms of action of clinical ABL kinase inhibitors. Nat Biotechnol. 2007; 25:10351044.
**PP2**	Selective inhibitor of Src-family tyrosine kinases	**Bantscheff M**, Eberhard D, Abraham Y, Bastuck S, Boesche M, Hobson S, Mathieson T, Perrin J, Raida M, Rau M, Reader V, Sweetman G, Bauer A, Bouwmeester T, Hopf C, et al. Quantitative chemical proteomics reveals mechanisms of action of clinical ABL kinase inhibitors. Nat Biotechnol. 2007; 25:10351044.
**SU11652**	Inhibitor that exhibits selectivity for PDGFRβ (IC_{50} = 3 nM), VEGFR2 (IC_{50} = 27 nM), and KIT family (IC_{50} = 10–500 nM)	**Ellegaard AM**, Groth-Pedersen L, Oorschot V, Klumperman J, Kirkegaard T, Nylandsted J, Jäättelä M. Sunitinib and SU11652 inhibit acid sphingomyelinase, destabilize lysosomes, and inhibit multidrug resistance. Mol Cancer Ther. 2013; 12:2018–2030.
**Curcumin**	TCf4/β-catenin interaction, Scavenger of ROS, anti-inflammatory	**Gupta SC**, Patchva S, Aggarwal BB. Therapeutic roles of curcumin: lessons learned from clinical trials. AAPS J. 2013; 15:195–218.
**Ponatinib**	Potent inhibitor of Abl, PDGFRα, VEGFR2, FGFR1 and Src with IC_{50} of 0.37 nM, 1.1 nM, 1.5 nM, 2.2 nM and 5.4 nM respectively	**Fauster A**, Rebsamen M, Huber KVM, Bigenzahn JW, Stukalov A, Lardeau CH, Scorzoni S, Bruckner M, Gridling M, Parapatics K, Colinge J, Bennett KL, Kubicek S, Krautwald S, Linkermann A, et al. A cellular screen identifies ponatinib and pazopanib as inhibitors of necroptosis. *Cell Death Dis*. 2015; 6:e1767.
**AZD5582**	SMAC mimetic and potent and selective inhibitor of XIAP and cellular cIAP	**Hennessy EJ**, Adam A, Aquila BM, Castriotta LM, Cook D, Hattersley M, Hird AW, Huntington C, Kamhi VM, Laing NM, Li D, MacIntyre T, Omer CA, Oza V, Patterson T, et al. Discovery of a novel class of dimeric Smac mimetics as potent IAP antagonists resulting in a clinical candidate for the treatment of cancer (AZD5582). J Med Chem. 2013; 56:9897–9919.
**5Z-7-Oxozeaenol**	Irreversible inhibitor of ERK2 (IC_{50} = 80 nM), TAK1, MKK7, and MEK1; anti-inflammatory	**Singh A**, Sweeney MF, Yu M, Burger A, Greninger P, Benes C, Haber DA, Settleman J. TAK1 inhibition promotes apoptosis in KRAS-dependent colon cancers. Cell. 2012; 148:639–650.
**Niclosamide**	Inhibitor of STAT3 signalling, mitochondrial uncoupler	**Li Y**, Li PK, Roberts MJ, Arend RC, Samant RS, Buchsbaum DJ. Multi-targeted therapy of cancer by niclosamide: A new application for an old drug. Cancer Lett. 2014; 349:8–14. **Tao H**, Zhang Y, Zeng X, Shulman GI, Jin S. Niclosamide ethanolamine-induced mild mitochondrial uncoupling improves diabetic symptoms in mice. Nat Med. 2014; 20:1263–1269. doi:10.1038/nm.3699.
**Rottlerin**	Mitochondrial uncoupler	**Soltoff SP**, Rottlerin is a mitochondrial uncoupler that decreases cellular ATP levels and indirectly blocks protein kinase Cδ tyrosine phosphorylation. J Biol Chem. 2001; 276:37986–37992.
**Rotenone**	Mitochondrial electron transport chain inhibitor (IC_{50} = 1.7–2.2 μM at complex I)	**Porporato PE**, Payen VL, Pérez-Escuredo J, De Saedeleer CJ, Danhier P, Copetti T, Dhup S, Tardy M, Vazeille T, Bouzin C, Feron O, Michiels C, Gallez B, Sonveaux P. A Mitochondrial Switch Promotes Tumor Metastasis. CellReports. 2014; 8:754–766.

Supplementary Table 2: Selective compounds that were identified as potential activators of mitochondrial fission

Activator	Putative targets	References
**Sanguinarine chloride**	Potent, noncompetitive Na^+/K^+-and Mg^{2+}-ATPase inhibitor, inhibitor of protein phosphatase 2C (PP2C)	**Gu S**, Yang XC, Xiang XY, Wu Y, Zhang Y, Yan XY, Xue YN, Sun LK, Shao GG. Sanguinarine-induced apoptosis in lung adenocarcinoma cells is dependent on reactive oxygen species production and endoplasmic reticulum stress. Oncol. Rep. 2015; 34:913–919.
**Plumbagin**	Inhibition of the Akt/mTOR pathway, induces G2/M cell cycle arrest and apoptosis in A549 cells through JNK-dependent p53 Ser15 phosphorylation, activation of Nrf2/ARE pathway	**Qiu JX**, Zhou ZW, He ZX, Zhao RJ, Zhang X, Yang L, Zhou SF, Mao ZF. Plumbagin elicits differential proteomic responses mainly involving cell cycle, apoptosis, autophagy, and epithelial-to-mesenchymal transition pathways in human prostate cancer PC-3 and DU145 cells. Drug Des Devel Ther. 2015; 9:349–417.
**A23187**	Ca^{2+} ionophore that increases intracellular Ca^{2+} concentration, inhibitor of mitochondrial ATPase activity, ER stress inducer	**Ding WX**, Ni HM, Gao W, Hou YF, Melan MA, Chen X, Stolz DB, Shao ZM, Yin XM. Differential effects of endoplasmic reticulum stress-induced autophagy on cell survival. J Biol Chem. 2007; 282:4702–4710.
**Tunicamycin**	ER stress inducer, induces G1 cell cycle arrest	**Ding WX**, Ni HM, Gao W, Hou YF, Melan MA, Chen X, Stolz DB, Shao ZM, Yin XM. Differential effects of endoplasmic reticulum stress-induced autophagy on cell survival. J Biol Chem. 2007; 282:4702–4710.
**Thapsigargin**	Potent inhibitor of sarco-endoplasmic reticulum Ca^{2+}-ATPases, ER stress inducer	**Ding WX**, Ni HM, Gao W, Hou YF, Melan MA, Chen X, Stolz DB, Shao ZM, Yin XM. Differential effects of endoplasmic reticulum stress-induced autophagy on cell survival. J Biol Chem. 2007; 282:4702–4710.
**Ionomycin**	Potent, highly selective Ca^{2+} ionophore that increases intracellular calcium concentrations, ER stress inducer	**Ji WK**, Hatch AL, Merrill RA, Strack S, Higgs HN, Lappalainen P. Actin filaments target the oligomeric maturation of the dynamin GTPase Drp1 to mitochondrial fission sites. eLife. 2015; 4:e11553.
**Dual Akt/ PDK1 inhibitor**	Inhibits both PDK1 and Akt activities in *in vitro* kinase assays in a dose-dependent manner	**Zeng Z**, Samudio IJ, Zhang W, Estrov Z, Pelicano H, Harris D, Frolova O, Hail N, Chen W, Kornblau SM, Huang P, Lu Y, Mills GB, Andreeff M, Konopleva M. Simultaneous Inhibition of PDK1/AKT and Fms-Like Tyrosine Kinase 3 Signaling by a Small-Molecule KP372-1 Induces Mitochondrial Dysfunction and Apoptosis in Acute Myelogenous Leukemia. Cancer Res. 2006; 66:3737–3746.
**NO-ASA**	Modulator of TCf4/β-catenin interaction	**De Santo C**, Serafini P, Marigo I, Dolcetti L, Bolla M, Del Soldato P, Melani C, Guiducci C, Colombo MP, Iezzi M, Musiani P, Zanovello P, Bronte V. Nitroaspirin corrects immune dysfunction in tumor-bearing hosts and promotes tumor eradication by cancer vaccination. PNAS. 2005; 102:4185–4190. **Nath N**, Vassell R, Chattopadhyay M, Kogan M, Kashfi K. Nitro-aspirin inhibits MCF-7 breast cancer cell growth: effects on COX-2 expression and Wnt/beta-catenin/TCF-4 signaling. Biochem. Pharmacol. 2009; 78:1298–1304.
